# Joint Replacement Versus Trapeziectomy for Trapeziometacarpal Osteoarthritis: A Systematic Review

**DOI:** 10.7759/cureus.94908

**Published:** 2025-10-19

**Authors:** Sunandan Datta, Bratati Bandyopadhyay, Muhammad Tahir, Rahul Shah, Mahak Baid, Mohammed Wahaaj Hussain, Krishnakumar Subbaraman

**Affiliations:** 1 Trauma and Orthopaedics, Aneurin Bevan University Health Board, Newport, GBR; 2 Orthopaedics, East Kent Hospitals University NHS Foundation Trust, Margate, GBR; 3 Orthopaedics and Traumatology, Aneurin Bevan University Health Board, Newport, GBR; 4 Trauma and Orthopaedics, Queen Elizabeth Hospital Birmingham, Birmingham, GBR

**Keywords:** hand surgeon, thumb carpometacarpal arthritis, thumb carpometacarpal joint, thumb cmc arthroplasty, thumb pain

## Abstract

Trapeziometacarpal (TMC) osteoarthritis is a common and disabling condition affecting hand function and quality of life. When conservative management fails, surgical intervention is warranted. The two primary surgical options are trapeziectomy, often combined with ligament reconstruction and tendon interposition (LRTI), and total joint arthroplasty using prosthetic implants. The objective of our study is to compare the efficacy, functional outcomes, patient satisfaction, and complication profiles of trapeziectomy versus joint replacement for TMC osteoarthritis. This review was conducted in accordance with Preferred Reporting Items for Systematic Reviews and Meta-Analyses (PRISMA) 2020 guidelines and specifically evaluates outcomes associated with the MAÏA™ (Groupe Lépine, France) and Touch® (KERIMEDICAL, Switzerland) prostheses compared to various trapeziectomy techniques. 11 studies, including retrospective and prospective cohort studies, a randomised controlled trial, and a same-patient comparison study, were analysed. Outcomes assessed included pain relief, grip and pinch strength, range of motion (ROM), patient-reported outcome measures (PROMs), recovery time, and complication rates. Both procedures effectively relieve pain and improve function. Joint replacement demonstrated superior short-term functional outcomes and faster recovery, particularly in strength restoration. However, it was associated with implant-specific complications. Trapeziectomy, while slower in recovery, showed consistent long-term efficacy and fewer implant-related issues. Both surgical options are viable. The choice should be individualised based on patient goals, anatomical considerations, and the balance between rapid functional recovery and implant-related risks.

## Introduction and background

Trapeziometacarpal (TMC) osteoarthritis, also known as rhizoarthrosis, is the second most common site of osteoarthritis in the hand, with a prevalence of up to 20% in individuals over 55 years of age [1]. In those over 70, the prevalence may reach 30% [2]. Radiographic studies show that thumb base osteoarthritis affects 5.8% of 50-year-old males and 7.3% of females, increasing to 33.1% and 39%, respectively, by age 80. The overall risk is 30% higher in females [3].

The TMC joint is essential for thumb mobility, particularly for pinch and grasp functions. Degeneration of the joint leads to pain, weakness, and functional impairment, often manifesting as restricted thumb adduction and a narrowing of the first web space [4].

When conservative treatments (e.g., splinting, nonsteroidal anti-inflammatory drugs (NSAIDs), corticosteroid injections) fail, surgical options include trapeziectomy and total joint arthroplasty. Trapeziectomy involves the excision of the trapezium bone, often combined with ligament reconstruction (LR) or tendon interposition (TI) using the flexor carpi radialis tendon. Techniques such as the Weilby suspensionplasty aim to prevent metacarpal subsidence. Although durable, trapeziectomy may result in a prolonged recovery and reduced thumb length, potentially affecting pinch strength [5-9].

Trapeziometacarpal joint arthroplasty (TMA) aims to restore joint anatomy and motion using prosthetic implants. Its advantages include the preservation of thumb length, improved biomechanics, and a faster return to function. The MAÏA™ (Groupe Lépine, France) and Touch® (KERIMEDICAL, Switzerland) prostheses are widely used in Europe and North America. These implants represent third- and fourth-generation designs developed to enhance stability, preserve motion, and promote a faster recovery. This review focuses on these two prostheses, comparing them to trapeziectomy to guide evidence-based surgical decision-making [10-13].

## Review

Materials and methods

This systematic review was conducted in accordance with Preferred Reporting Items for Systematic Reviews and Meta-Analyses (PRISMA) 2020 guidelines to evaluate clinical outcomes of trapeziectomy versus total joint replacement using MAÏA or Touch prostheses for TMC osteoarthritis.

Search Strategy

The protocol was registered on PROSPERO (CRD420251137542). A structured search using the terms “Maia*” AND “trapeziectom*” and “Touch*” AND “trapeziectom*” was performed across MEDLINE (13), Embase (30), Emcare (7), CINAHL (0), BNI (0), Cochrane Library (5), and TRiP (3). After deduplication in EndNote Web, 32 records were screened. Eleven studies met inclusion criteria.

Eligibility Criteria

Included studies involved adult patients with primary TMC osteoarthritis undergoing trapeziectomy or joint replacement using MAÏA or Touch prostheses. Minimum follow-up was 12 months. Eligible designs included RCTs, cohort studies, and comparative observational studies (Levels I-IV).

Exclusion criteria involved non-surgical management, pediatric populations, concomitant hand surgeries, revision procedures, isolated ligament/tendon procedures, arthrodesis, hematoma distraction arthroplasty, non-standard techniques, case reports, expert opinions, narrative reviews, conference abstracts, and non-English publications without translation.

Interventions and Comparators

Intervention: Total joint arthroplasty using MAÏA or Touch prostheses.

Comparator: Trapeziectomy, including simple excision, TI, LR, ligament reconstruction and tendon interposition (LRTI), and resection-suspension arthroplasty (RSA).

Data Extraction and Risk of Bias 

Two reviewers independently extracted data. Variables included study design, year, sample size, demographics, surgical technique, follow-up duration, level of evidence, and outcome measures. Discrepancies were resolved by consensus.

Risk of bias was substantial. Only the RCT by Guzzini et al. and the within-patient study by Nietlispach et al. showed low to moderate risk. The remaining studies exhibited high or critical risk due to selection bias and confounding.

Outcomes 

Primary outcomes: Pain relief (Visual Analog Scale (VAS)), functional scores (Quick Disabilities of the Arm, Shoulder, and Hand (QuickDASH), Michigan Hand Outcomes Questionnaire (MHQ), Kapandji), grip/pinch strength.

Secondary outcomes: Complication rates, revision frequency, implant survival, long-term durability, recovery time, return to work.

**Figure 1:**
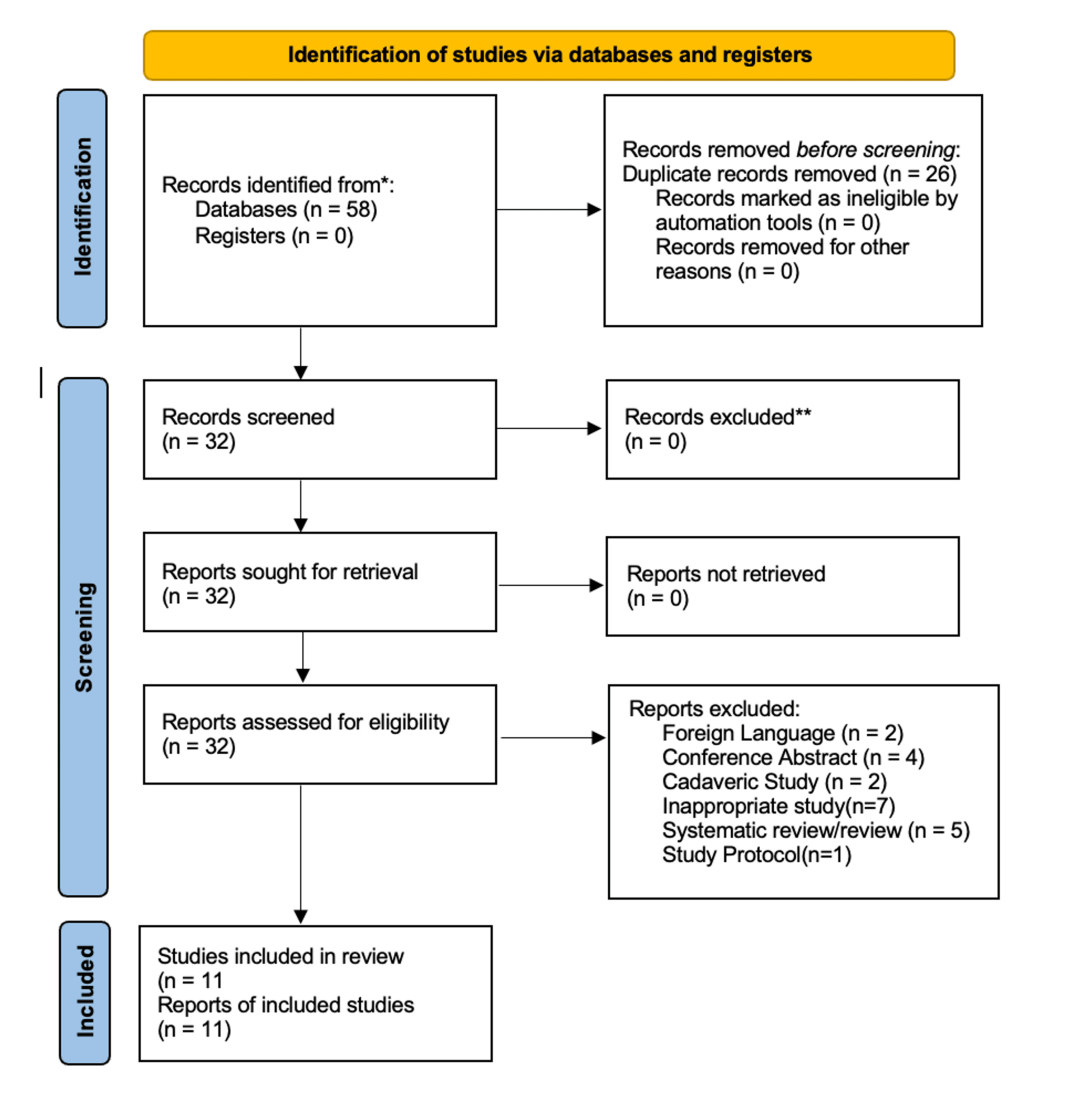
PRISMA 2020 flow diagram of the study selection process PRISMA: Preferred Reporting Items for Systematic Reviews and Meta-Analyses

The PRISMA flow diagram is illustrated in Figure 1 below.

The summary of included studies is represented in Table 1 below.

**Table 1:** Summary of included studies This table summarizes the key demographic, design, and outcome characteristics of the 11 studies included in the systematic review. PROM: Patient-reported outcome measure; ROM: Range of motion; QuickDASH, Quick Disabilities of the Arm, Shoulder, and Hand; MHQ: Michigan Hand Outcomes Questionnaire; RSIA: Resection-suspension interposition arthroplasty; NRS: Numeric Rating Scale; VAS: Visual Analog Scale; TI: Tendon interposition

Name of Study	Authors	Year (Published)	Year of Investigation	Total Patients	Follow-up (months)	Procedure Type	Mean Age (years)	Level of Evidence	PROMs / Functional Outcomes
Complications and failures of the trapeziometacarpal Maia prosthesis: A series of 156 cases [14]	Bricout & Rezzouk	2016	2010–2015	139 (156 thumbs)	37.8	MAÏA prosthesis	62.7	IV	Pain, ROM, strength, complications; no standardised PROM
Failure Rate and Early Complications of Thumb Carpometacarpal Joint Replacement – A Multicenter Retrospective Study of Two Modern Implant Designs [15]	Farkash et al.	2024	2015–2022	381	36	MAÏA and Touch prostheses	63 ± 9	III	QuickDASH, MHQ, pain, strength, satisfaction
Touch Double Mobility Arthroplasty for Trapeziometacarpal Osteoarthritis: Outcomes for 92 Prostheses [16]	Gonzalez-Espino et al.	2021	2016–2019	92	16	Touch 1 dual mobility prosthesis	62.2 ± 7.9	IV	MHQ, QuickDASH, pain, ROM, strength
Interposition Arthroplasty versus Dual Cup Mobility Prosthesis in Treatment of Trapeziometacarpal Joint Osteoarthritis: A Prospective Randomized Study [17]	Guzzini et al.	2023	2018–2020	139 (150 hands)	24	Touch prosthesis vs. TI arthroplasty	~67	I	QuickDASH, MHQ, pain, strength, satisfaction
Low Complication Rate and High Implant Survival at 2 Years after Touch Trapeziometacarpal Joint Arthroplasty [18]	Herren et al.	2023	2018–2020	130	24	Touch VR dual mobility prosthesis	63 ± 8	III	QuickDASH, MHQ, pain, ROM, strength
Which Would You Choose Again? Comparison of Trapeziometacarpal Implant versus Resection Arthroplasty in the Same Patient [19]	Nietlispach et al.	2024	2019–2022	14	26.4	Touch VR prosthesis vs. RSIA	62 (range 54–84)	IV	MHQ, pain, ROM, strength
Management of the Capsule in Trapeziometacarpal Joint Implant Arthroplasty: Resection versus Repair [20]	Reischenböck et al.	2024	2018–2022	188	12	Touch VR prosthesis (capsule resection vs repair)	62–63	III	Brief MHQ, Kapandji score, pain (NRS), key pinch strength
Comparison between the MAIA® Implant and Trapeziectomy for Trapeziometacarpal Osteoarthritis: Outcomes at 9 Years’ Follow-Up [21]	Seaourt et al.	2021	2009–2011	92	108	MAÏA prosthesis vs. trapeziectomy	57	III	QuickDASH, MHQ, pain (VAS), ROM, strength
Comparative Study of Trapeziectomy with Weilby Suspensionplasty versus Implant Arthroplasty for Thumb Carpometacarpal Joint Arthritis in an Asian Population [22]	Tan & Kang	2024	2015–2023	13 (15 thumbs)	14 (implant), 4.5 (Weilby)	Touch prosthesis vs. trapeziectomy + Weilby suspensionplasty	62–63	III	Kapandji score, pain (NRS), grip/pinch strength, return to work
Trapeziectomy versus Maïa Prosthesis in Trapeziometacarpal Osteoarthritis [23]	Windhofer et al.	2024	2018–2021	58 (59 thumbs)	12	MAÏA prosthesis vs. Weilby TI	59 (prosthesis), 64 (Weilby)	IV	Modified Mayo wrist score, pain (Alnot/Saint Laurent), key pinch strength, return to work
MAÏA Trapeziometacarpal Joint Arthroplasty: Clinical and Radiological Outcomes of 76 Patients With More Than 10 Years of Follow-Up [24]	Toffoli et al.	2024	2006–2009	76 (92 implants)	134	MAÏA prosthesis (long-term follow-up)	67	IV	QuickDASH, VAS, Kapandji score, grip/pinch strength, ROM, return to work

Results

This systematic review analysed data from 11 studies comparing trapeziectomy and total joint arthroplasty using MAÏA™ or Touch® prostheses for the management of TMC osteoarthritis. The outcomes were categorized into primary and secondary domains, focusing on pain relief, functional recovery, grip and pinch strength, complication rates, implant survival, and return to work.

Primary Outcomes

Pain Relief: Both procedures resulted in significant pain reduction. Guzzini et al. reported statistically significant improvements in pain for both the trapeziectomy and Touch® prosthesis groups at all follow-ups, with a faster early reduction in the implant group [17]. Similarly, Windhofer et al. found that patients with the MAÏA prosthesis reported significantly less pain at three months (median VAS 1.0 vs. 2.0, p=0.04) [23]. However, this difference between groups was not significant at the one-year mark [23]. In a long-term follow-up of nine years, Seaourt et al. found no significant difference in final pain levels between the arthroplasty and trapeziectomy groups [21].

Functional Outcomes: Functional scores, assessed with standardised questionnaires, generally showed an early advantage for joint replacement. Guzzini et al. observed a faster improvement in functional scores for the prosthesis group [17]. In a within-patient comparison, Nietlispach et al. reported significantly higher MHQ scores for the hand that received an implant versus the one that had a trapeziectomy (median 88.5 vs. 66.7, p<0.05) [19]. At nine years, however, Seaourt et al. found no significant difference in QuickDASH scores between the two procedures [21]. In a non-comparative study, Toffoli et al. reported that the MAÏA prosthesis led to a significant improvement in QuickDASH scores, from 61.3 preoperatively to 19.6 postoperatively (p<0.001) [24].

Grip and Pinch Strength: Strength measurements consistently favoured the joint replacement groups. The within-patient study by Nietlispach et al. (2024) found significantly higher key pinch strength (6.5 kg vs. 4.5 kg, p=0.016) and grip strength (26.5 kg vs. 21.0 kg, p=0.006) in the implant group [19]. Seaourt et al. also reported better pinch strength in the implant group (mean 5.4 kg vs. 4.0 kg, p<0.05), though grip strength was comparable between groups [21]. Tan and Kang noted higher grip strength at three months in their arthroplasty group, but this difference was no longer significant at six months [22].

Secondary Outcomes

Complication and Revision Rates: Reported complication rates were higher for total joint arthroplasty. Bricout and Rezzouk documented a 35.9% overall complication rate and an 11.5% revision rate in a series of 156 MAÏA prostheses [14]. Farkash et al. found a 19.2% complication rate and a 10.8% reoperation rate for modern implants [15]. For the Touch® prosthesis, Herren et al. reported a 16.8% complication rate and a 3.1% revision rate [18]. In one study by Nietlispach et al., two revisions were reported in the trapeziectomy group, while the implant group had none [19].

Implant Survival and Durability: Long-term implant survival was reported in two studies. Toffoli et al. reported an 88% survival rate for the MAÏA prosthesis at a mean follow-up of 134 months [24]. The most common causes for failure were loosening (4.4%) and dislocation (2.2%) [24]. For the Touch® prosthesis, Herren et al. reported a 96% survival rate at two years [18].

Recovery Time and Return to Work: Patients who underwent joint replacement were found to return to activities sooner. Seaourt et al. reported a mean recovery time of six weeks in the implant group [21]. Tan and Kang found a median return-to-work time of four weeks for arthroplasty patients compared to six weeks for the trapeziectomy group [22].

Discussion

This systematic review demonstrates that both trapeziectomy and total joint arthroplasty are effective surgical options for managing end-stage TMC osteoarthritis. The evidence does not establish the clear superiority of one procedure over the other; instead, it highlights a trade-off between rapid functional recovery and long-term risks. Both approaches yield significant improvements in pain and function. However, arthroplasty offers earlier gains in strength and a quicker return to daily activities, advantages that are counterbalanced by a higher incidence of implant-related complications and revision surgeries. In contrast, trapeziectomy provides durable, reliable symptom relief with a lower revision burden, a finding consistent with the meta-analysis by Challoumas et al. [5].

A recurring theme across the included studies is the accelerated recovery associated with joint replacement. Patients receiving dual-mobility implants like the MAÏA™ or Touch® prostheses consistently demonstrate superior early improvements in grip and pinch strength, quicker return to work, and higher satisfaction scores [17,19,22]. The within-patient evidence from Nietlispach et al. is particularly compelling, showing enhanced thumb strength following arthroplasty in the same individual [19]. This functional advantage is likely attributable to the preservation of joint length and biomechanics with prosthetic implants, which avoids the thumb shortening often seen with trapeziectomy [19]. These findings are further supported by Gonzalez-Espino et al., who reported excellent outcomes with the Touch® prosthesis [16].

However, the short-term benefits of arthroplasty must be carefully weighed against its complication profile. Our review found complication rates for arthroplasty ranging from 15% to 35.9% and revision rates between 10% and 13%, reinforcing the higher risk profile associated with joint replacement [14,15,18]. Bricout et al. reported notable rates of prosthesis dislocation and loosening, while Farkash et al. identified early implant-related issues such as tenosynovitis [14,15]. These concerns are echoed in systematic reviews by Ganhewa et al. and Latelise et al., which also highlight significantly higher complication and revision rates for arthroplasty compared to trapeziectomy [25,26]. This underscores the critical importance of patient selection and thorough preoperative counselling when considering a joint replacement.

Trapeziectomy remains a reliable and definitive procedure, particularly for patients prioritising long-term durability and lower surgical risk. Although recovery may be slower, studies such as Seaourt et al. demonstrate comparable long-term outcomes in pain and function at nine years [21]. Crucially, trapeziectomy eliminates the risk of implant failure and serves as a dependable salvage option following a failed arthroplasty. While Windhofer et al. acknowledge the extended rehabilitation period as a drawback, its proven long-term reliability makes it an excellent standard of care [23].

The findings of this review align with those of Latelise et al., who reported a 6% complication rate for trapeziectomy compared to nearly 25% for arthroplasty, with revision rates of 2% and 13%, respectively [26]. Pooled data from the included studies revealed arthroplasty complication rates ranging from 15% to 35.9%, and revision rates between 10% and 13%, reinforcing the higher risk profile associated with joint replacement.

A 2012 study by Vandenberghe et al. found that thumb joint replacement (arthroplasty) has a higher risk of dislocation and re-operation compared to trapeziectomy. Given that joint replacement is also significantly more expensive, the authors recommended trapeziectomy as the first-line surgical treatment for thumb base arthritis [27].

Procedure selection should be individualised, taking into account patient age, activity level, and comorbidities. Younger, active individuals seeking rapid functional recovery may benefit from arthroplasty, provided they are informed of the associated risks. Conversely, older or less active patients may prefer the long-term reliability and lower complication rates of trapeziectomy. These considerations are echoed in the randomised trial by de Jong et al. and the systematic review by Raj et al., both of which emphasise the importance of thorough preoperative counselling and tailored patient selection [28,29].

Limitations

This review is limited by its reliance on a pre-selected set of studies, which may introduce selection bias and restrict comprehensiveness. The included studies exhibit methodological heterogeneity, variable follow-up durations, and differences in implant types, complicating direct comparisons. Notably, outcomes may be implant-specific, as suggested by differing complication profiles between the MAÏA™ and Touch® prostheses-an area that warrants further investigation. The absence of long-term head-to-head randomised trials limits the ability to draw definitive conclusions regarding procedural superiority. Additionally, subgroup analyses of arthroplasty types and trapeziectomy variants were not performed, which may obscure nuanced differences in outcomes. Although the literature search was conducted nearly a year prior to submission, the scarcity of high-quality studies in this domain underscores the relevance and value of this review in guiding surgical management of base-of-thumb osteoarthritis.

## Conclusions

In conclusion, both trapeziectomy and total joint arthroplasty are effective surgical treatments for thumb basal osteoarthritis. TMA provides a clear advantage in terms of faster recovery, earlier pain relief, and superior strength, making it an attractive option for younger, active, or working patients who prioritise a rapid return to function. However, these benefits come at the cost of higher rates of complications and the potential need for future revision surgery. Trapeziectomy remains a highly reliable and durable procedure with a lower risk profile, offering predictable long-term pain relief. The decision must be individualised through a shared decision-making process, carefully weighing the patient's age, activity level, and tolerance for risk against the distinct advantages and disadvantages of each approach.
